# Characteristics of occupational exposure associated with traditional Chinese medicine-related clinical procedures and comparison with non-TCM clinical practice: a single-center retrospective study

**DOI:** 10.3389/fpubh.2026.1918328

**Published:** 2026-09-10

**Authors:** Li Ma, Cheng Yang, Ling Ai, Jing Chen

**Affiliations:** 1 Mianyang Hospital of TCM, Mianyang, Sichuan, China; 2 Sichuan Institute of Industrial Technology, Deyang, Sichuan, China; 3 Sichuan College of Traditional Chinese Medicine, Mianyang, Sichuan, China

**Keywords:** infection prevention and control, needlestick injury, occupational exposure, retrospective study, sharps injury, traditional Chinese medicine nursing

## Abstract

**Objective:**

To analyze the characteristics of occupational exposure events reported in a tertiary hospital between 2022 and 2025, with a particular focus on occupational exposures associated with traditional Chinese medicine (TCM)-related clinical procedures, and to compare the characteristics of TCM-related and non-TCM occupational exposure events, thereby providing descriptive evidence to support the prevention and management of occupational exposure in TCM-related clinical practice.

**Methods:**

A retrospective study was conducted using occupational exposure records reported in a tertiary hospital between January 2022 and December 2025. A total of 188 occupational exposure events were included. According to whether the exposure occurred during TCM-related clinical procedures, the events were classified into a TCM-related occupational exposure group and a non-TCM occupational exposure group. Descriptive analyses were performed to summarize demographic characteristics, exposure characteristics, and post-exposure management. Exploratory comparisons between the two groups were conducted using appropriate statistical methods.

**Results:**

A total of 188 occupational exposure events were included, of which 48 (25.53%) were related to TCM clinical procedures. The overall number of occupational exposure events peaked in 2023 (55 cases), and the highest monthly number was observed in August (23 cases). Most exposed healthcare workers were female (74.47%) and had less than 5 years of work experience (76.06%). Sharps injuries accounted for the majority of occupational exposure events (87.77%). Among the reported TCM-related occupational exposure events, most occurred during acupuncture-related procedures and involved acupuncture needle injuries. Compared with non-TCM occupational exposure events, no statistically significant differences were observed in age, sex, year of occurrence, or indicators related to post-exposure management (all *p* > 0.05). Significant differences were observed in occupational role, exposure stage, exposure type, type of sharp instrument, injured body site, and glove use (all *p* < 0.05). Regarding post-exposure management, all cases received immediate wound irrigation and disinfection, and 99.47% were reported within 24 h. However, only 29.26% completed the recommended 6-month follow-up and testing protocol. No confirmed HBV, HCV, or HIV infections were identified among healthcare workers who completed the recommended follow-up and testing protocol.

**Conclusion:**

Sharps injuries accounted for the majority of reported occupational exposure events. TCM-related occupational exposure events exhibited distinct characteristics, particularly those involving acupuncture-related procedures and acupuncture needle injuries. Although compliance with immediate post-exposure management and reporting was high, completion of long-term follow-up and testing remained insufficient. These findings provide descriptive evidence that may help inform the optimization of occupational exposure prevention and post-exposure management for TCM-related clinical procedures.

## 1 Introduction

Occupational exposure refers to incidents in which healthcare workers come into contact with blood, body fluids, pathogenic microorganisms, or other hazardous factors during medical treatment, nursing care, and related healthcare activities, thereby posing potential health risks to themselves (1, 2). Such exposures not only increase the risk of transmission of blood-borne pathogens, including hepatitis B virus (HBV), hepatitis C virus (HCV), and human immunodeficiency virus (HIV) (3, 4), but may also impose considerable psychological stress on healthcare workers due to uncertainty regarding infection outcomes. Previous studies have demonstrated that occupational exposure is closely associated with increased levels of anxiety, fear, and occupational stress among healthcare personnel, which may subsequently compromise both occupational health and the quality of healthcare services (5). Therefore, occupational exposure has become an important issue in the fields of hospital infection control and occupational health management.

Previous studies have shown that occupational exposure is common across healthcare institutions at all levels, with sharps injuries representing one of the predominant exposure types. A systematic review and meta-analysis reported a pooled prevalence of 43.3% for occupational injuries among healthcare workers, with sharps injuries and exposure to contaminated body fluids being the most common forms of injury (6). Another systematic review and meta-analysis, including 87 studies involving 50,916 healthcare workers, demonstrated that the pooled one-year prevalence of needlestick injuries was 44.5%, and hollow-bore needles were identified as the most frequent causative device (55.1%) (7). Furthermore, a retrospective study involving 151 reported occupational exposure events found that sharps injuries accounted for 76.16% of all cases and primarily affected the hands. The study also described differences in exposure characteristics according to years of work experience (8). Occupational exposure events are frequently reported during clinical procedures such as injection, blood collection, surgical operations, and sharps handling. Previous studies have described variations in reported exposure characteristics according to occupational category, years of professional experience, working environment, adherence to standard operating procedures, and implementation of personal protective measures (9, 10). Previous survey-based studies have reported that the prevalence of needlestick injuries among healthcare workers ranges from approximately 43 to 58% across different regions. These studies have also suggested that less experienced healthcare workers may report a higher frequency of needlestick injuries compared with more experienced personnel (11). To date, however, most studies have focused on healthcare workers in occupations traditionally considered to have a high likelihood of occupational exposure, such as nurses, surgeons, and laboratory personnel, whereas comparatively little attention has been paid to differences in occupational exposure characteristics across distinct clinical practice settings.

In recent years, with the continuous promotion of the inheritance and innovative development of traditional Chinese medicine (TCM) in China, the capacity of TCM healthcare services has been steadily enhanced. A national survey reported that 84.27% of physicians incorporated integrated traditional Chinese and Western medicine into their clinical practice, while 69.39% routinely performed non-pharmacological TCM therapies such as acupuncture and tuina (12). Traditional external therapeutic techniques, including acupuncture, moxibustion, and bloodletting therapy, constitute important components of the TCM clinical system (13). Among these, acupuncture is one of the most widely practiced TCM interventions and is characterized by frequent manipulation of sharp instruments and repeated needle insertion, thereby exposing healthcare workers to potential occupational hazards throughout clinical practice. Existing evidence suggests that acupuncture-related occupational exposure is not uncommon. A survey conducted in a tertiary TCM hospital reported that 63 of 5,069 healthcare workers engaged in acupuncture-related procedures reported accidental needlestick injuries, corresponding to a self-reported occurrence proportion of 1.24% (14). In another cross-sectional study involving 578 acupuncture practitioners from 98 hospitals in China, 34.3% of respondents reported experiencing at least one needlestick injury during the previous three years, while 46.0% of these incidents were not officially reported (15). These findings indicate that acupuncture-related occupational exposure is relatively common in clinical practice and is accompanied by substantial underreporting. Overall, compared with conventional clinical procedures, TCM-related therapeutic techniques involve unique types of sharp instruments, procedural workflows, and exposure scenarios, suggesting that the characteristics and patterns of reported occupational exposure events associated with TCM-related procedures may differ from those observed in conventional clinical settings and warrant further investigation.

Therefore, this study retrospectively analyzed occupational exposure records collected from a tertiary hospital between 2022 and 2025. The study aimed to describe the characteristics of reported occupational exposure events, with particular attention to TCM-related occupational exposure events, and to compare reported TCM-related and non-TCM-related occupational exposure events regarding demographic characteristics, exposure settings, exposure procedures, exposure types, and post-exposure management. The findings are expected to provide descriptive evidence to inform occupational safety management of TCM-related clinical procedures and support the optimization of occupational exposure prevention and post-exposure management strategies.

## 2 Methods

### 2.1 Study design and study population

This study adopted a single-center retrospective observational design. Data were obtained from occupational exposure incident reports recorded in the occupational exposure management system of a tertiary Grade A hospital between January 2022 and December 2025. The aim was to analyze the epidemiological characteristics of occupational exposure events, describe the distribution patterns of traditional Chinese medicine (TCM)-related occupational exposures, and compare differences between TCM-related and non-TCM occupational exposure events. The occupational exposure management system records reported exposure events but does not routinely capture denominator data, such as the annual number of healthcare workers, the total number of clinical procedures, or the number of TCM-related procedures performed. Therefore, this study was designed to describe and compare reported occupational exposure events rather than to estimate the incidence or quantify the risk of occupational exposure.

The inclusion criteria were as follows: (1) occupational exposure incidents reported and registered in the hospital occupational exposure management system during the study period; and (2) records with relatively complete information, allowing identification of exposure type and post-exposure management. The exclusion criteria were: (1) duplicate or repeated reports of the same exposure event; and (2) records with missing key variables (e.g., exposure type or post-exposure management) that could not be retrieved from the original documentation.

According to the clinical context in which exposure occurred, participants were categorized into a TCM-related occupational exposure group and a non-TCM occupational exposure group. TCM-related occupational exposure was defined as any exposure event occurring during TCM-specific diagnostic or therapeutic procedures, including acupuncture, electroacupuncture, and other needle-based interventions, in which sharps injuries or related exposure events occurred. The unit of analysis was individual reported occupational exposure events. Review of the original records confirmed that the 188 reported occupational exposure events involved 188 unique healthcare workers, with no repeated occupational exposure events reported for the same individual during the study period. Therefore, each reported event was treated as one independent analytical observation.

**Figure 1:**
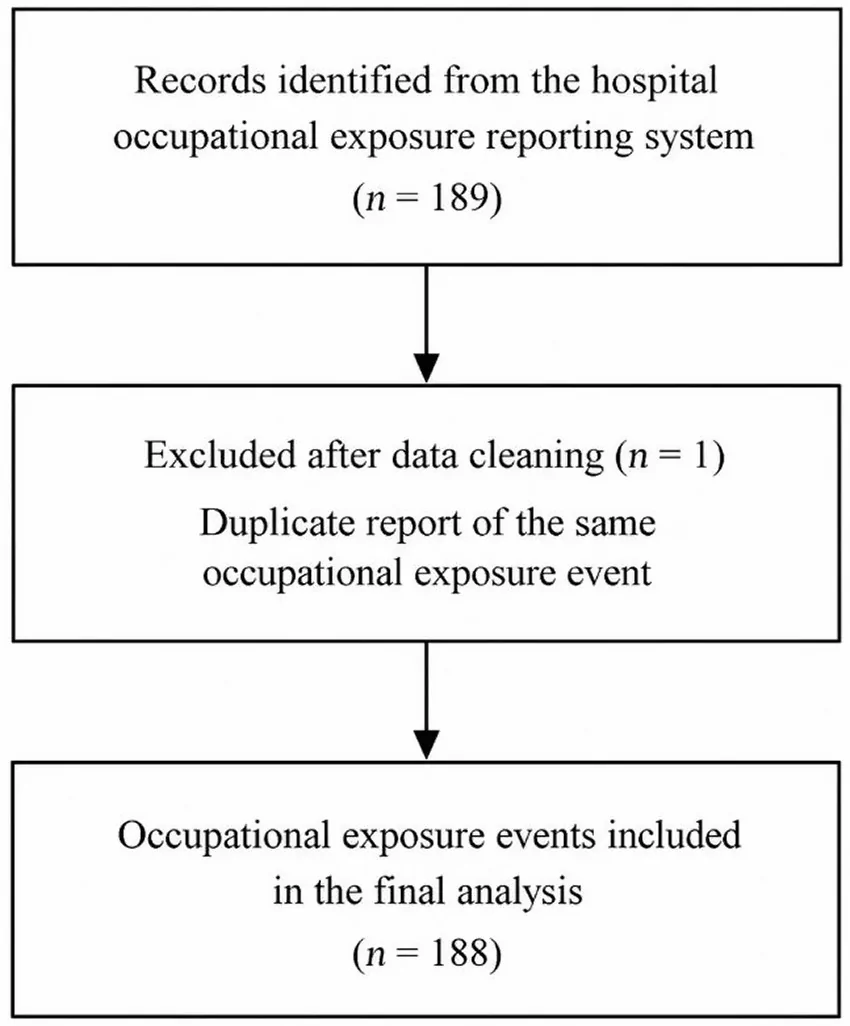
Flow diagram of occupational exposure record selection.

A total of 189 occupational exposure records were initially identified from the hospital occupational exposure reporting system. During data cleaning, one duplicate report of the same occupational exposure event was excluded. Finally, 188 occupational exposure events were included in the final analysis (Figure 1).

### 2.2 Data collection and variable definition

Occupational exposure data were extracted through a systematic review of the hospital occupational exposure registry system and related occupational exposure management databases. The collected information included the following categories: (1) Demographic and occupational characteristics: year of occurrence, age, sex, years of service, and professional position; (2) Exposure-related characteristics: location of exposure, procedure stage during which the exposure occurred, type of exposure, type of sharp instrument involved, injured body site, and use of personal protective measures (e.g., glove use); (3) Source-related information: results of source patient testing and infection-related status when available; (4) Post-exposure management: whether immediate wound washing was performed, whether disinfection measures were applied, whether the incident was reported within 24 h, whether post-exposure prophylaxis was administered, and whether a 6-month follow-up and testing protocol was completed. According to the Guideline for the Protection Against Occupational Exposure to Blood-borne Pathogens issued by the (23) and the corresponding occupational exposure management protocol implemented in our hospital, all healthcare workers experiencing occupational exposure were routinely scheduled for standardized follow-up for six months, regardless of the exposure source status or whether post-exposure prophylaxis (PEP) was indicated. Standardized follow-up included baseline serological testing immediately after exposure and repeat laboratory examinations at 3 and 6 months after exposure. Completion of follow-up was defined as completion of all scheduled examinations throughout the six-month follow-up period. Therefore, all occupational exposure events included in this study were considered eligible for follow-up. In contrast, the administration of PEP was determined individually according to the exposure type, source-patient infection status, immune status of the exposed healthcare worker, and the hospital occupational exposure management protocol; (5) Exposure outcomes: occurrence of blood-borne infections, including hepatitis B virus (HBV), hepatitis C virus (HCV), and human immunodeficiency virus (HIV). Occupational exposure events were categorized into three types: sharps injuries, blood/body fluid exposure, and other exposure types.

### 2.3 Definition of TCM-related occupational exposure

In this study, TCM-related occupational exposure was defined as any occupational exposure incident occurring during the performance of traditional Chinese medicine (TCM)–specific clinical procedures. These procedures included acupuncture, electroacupuncture, acupoint-based therapies, and other needle-related interventions, in which exposure events involved sharps injuries or other forms of occupational exposure.

### 2.4 Quality control

To ensure data accuracy and reliability, all occupational exposure records were independently extracted and reviewed by two trained researchers. Any discrepancies or uncertain entries were resolved through re-examination of the original registry records until consensus was achieved. After data entry, logical consistency checks and duplicate verification were performed to minimize information bias and data entry errors.

### 2.5 Statistical analysis

Statistical analyses were performed using SPSS version 26.0. Normally distributed continuous variables were expressed as mean ± standard deviation (x̄ ± s), and comparisons between groups were conducted using independent-samples t-tests. Categorical variables were presented as frequencies and percentages. Pearson’s chi-square test was used when the assumptions for the chi-square test were satisfied. Fisher’s exact test was applied for sparse 2 × 2 contingency tables, whereas the Fisher–Freeman–Halton exact test with Monte Carlo simulation was used for larger contingency tables containing small expected cell counts or zero observations. For variables with missing values, available-case analysis was performed, whereby cases with missing data were excluded only from analyses involving the corresponding variable. No imputation or sensitivity analyses were performed because the primary objective of this study was descriptive. Comparisons between TCM-related and non-TCM-related occupational exposure events were exploratory and intended to describe differences in event characteristics rather than to test prespecified hypotheses. Therefore, no formal adjustment for multiple comparisons was applied. A two-sided *p* value < 0.05 was considered statistically significant.

### 2.6 Ethical approval

This study was approved by the Medical Ethics Committee of the hospital (approval no. 2026-38). As this was a retrospective study and did not involve patient-identifiable information or any intervention, the requirement for informed consent was waived by the ethics committee.

## 3 Results

### 3.1 Overall characteristics of occupational exposure events

**Figure 2:**
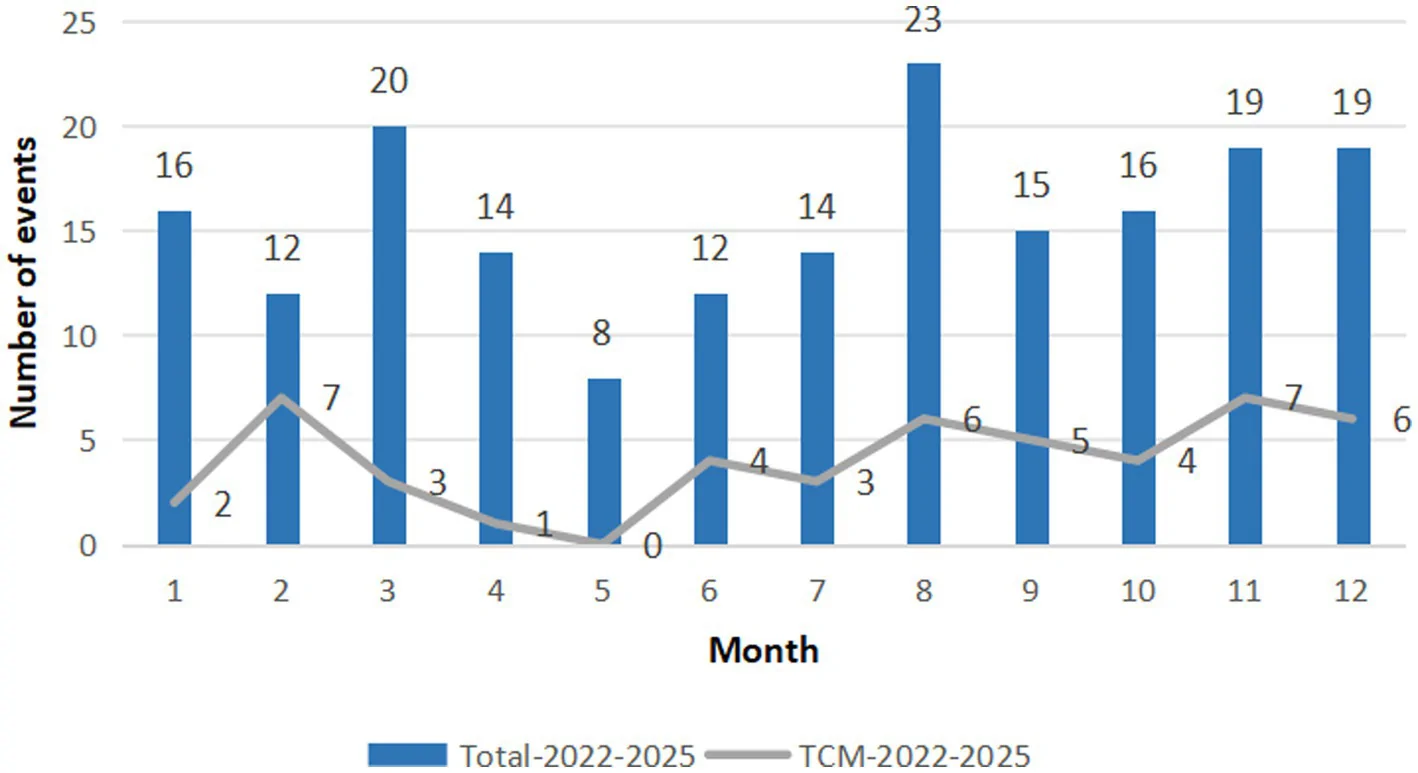
Monthly distribution of occupational exposure events from 2022 to 2025.

**Figure 3:**
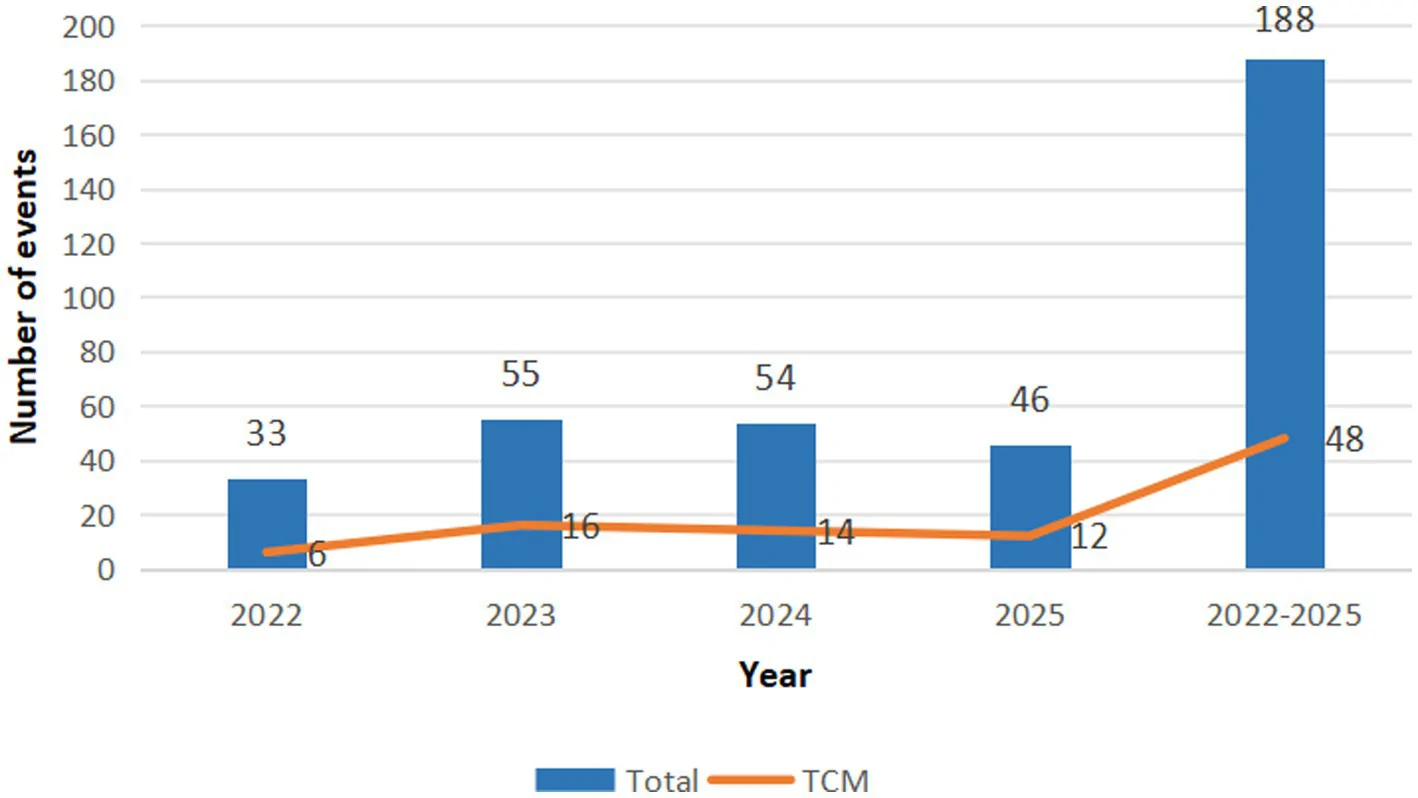
Annual trend of occupational exposure events from 2022 to 2025.

The annual distribution of reported occupational exposure events showed the largest number of reported events in 2023 (*n* = 55), followed by a slight decline thereafter. Similarly, the largest number of reported TCM-related occupational exposure events was observed in 2023 (*n* = 16). Reported occupational exposure events occurred throughout the year, with the largest number recorded in August (*n* = 23) and the smallest number in May (*n* = 8). Because denominator information, such as staffing levels and procedure volume, was unavailable, these findings describe the distribution of reported occupational exposure events only and should not be interpreted as reflecting temporal changes in occupational exposure risk. Among the reported TCM-related occupational exposure events, the highest numbers were observed in February and November (*n* = 7 each) (Figures 2, 3).

Among the 188 exposed healthcare workers, the mean age was 27.96 ± 7.15 years, and 74.47% were female. Individuals with less than 5 years of work experience accounted for 76.06%. In terms of occupational composition, interns, staff nurses, and physicians accounted for 24.47, 21.28, and 16.49%, respectively. Most exposures occurred in inpatient wards (48.40%). The main exposure-related procedures were injection/puncture (27.13%), needle removal (26.06%), and sharps handling/disposal (19.68%). Sharps injuries were the predominant exposure type (87.77%), with the hands being the most frequently affected site (73.94%). Regarding protective measures, 67.02% of individuals were not wearing gloves at the time of exposure. All cases received immediate wound irrigation and disinfection, and 99.47% were reported within 24 h. In terms of post-exposure prophylaxis, most healthcare workers did not receive medication, whereas only a small proportion received prophylactic treatment. Although immediate post-exposure management was generally well implemented, completion of the recommended 6-month follow-up and testing protocol remained relatively low, resulting in a substantial proportion of indeterminate infection outcomes. Detailed information is presented in Table 1.

**Table 1:** Analysis of occupational exposure events from 2022 to 2025.

Variables	Items	Overall occupational exposure (*N* = 188), *n* (%)	TCM-related occupational exposure (*N* = 48), *n* (%)
Year	2022	33 (17.55)	6 (12.50)
	2023	55 (29.26)	16 (33.33)
	2024	54 (28.72)	14 (29.17)
	2025	46 (24.47)	12 (25.00)
Sex	Female	140 (74.47)	32 (66.67)
	Male	48 (25.53)	16 (33.33)
Years of service	<5	143 (76.06)	40 (83.33)
	5–10	15 (7.98)	4 (8.33)
	10–20	26 (13.83)	3 (6.25)
	≥20	4 (2.13)	1 (2.08)
Occupational role	Intern nurse	18 (9.57)	0 (0.00)
	Intern technician	5 (2.66)	1 (2.08)
	Intern physician	46 (24.47)	24 (50.00)
	Resident nurse	16 (8.51)	2 (4.17)
	Resident physician	19 (10.11)	9 (18.75)
	Advanced training physician	3 (1.60)	2 (4.17)
	Employed worker	6 (3.19)	1 (2.08)
	Staff nurse	40 (21.28)	5 (10.42)
	Staff technician	4 (2.13)	0 (0.00)
	Staff physician	31 (16.49)	4 (8.33)
Location of occurrence	Treatment room	33 (17.55)	8 (16.67)
	Operating room	15 (7.98)	0 (0.00)
	Therapy room	12 (6.38)	7 (14.58)
	Ward	91 (48.40)	28 (58.33)
	Outpatient clinic	21 (11.17)	5 (10.42)
	Others	16 (8.51)	0 (0.00)
Exposure process	Needle removal/withdrawal	49 (26.06)	25 (52.08)
	Instrument cleaning and handling	20 (10.64)	0 (0.00)
	Surgical/suturing procedure	14 (7.45)	0 (0.00)
	Injection/puncture procedure	51 (27.13)	2 (4.17)
	Special exposure events	1 (0.53)	0 (0.00)
	Environmental/residual sharp exposure	1 (0.53)	1 (2.08)
	Electroacupuncture/acupuncture procedure	12 (6.38)	12 (25.00)
	Blood/body fluid exposure	3 (1.60)	0 (0.00)
	Sharp disposal/sharps handling	37 (19.68)	8 (16.67)
Type of exposure	Blood/body fluid exposure	22 (11.70)	0 (0.00)
	Sharps injury	165 (87.77)	48 (100.00)
	Other	1 (0.53)	0 (0.00)
Sharp instrument type	None	23 (12.23)	0 (0.00)
	Medical device	14 (7.45)	1 (2.08)
	Surgical needle	15 (7.98)	0 (0.00)
	Injection/blood collection needle	91 (48.40)	2 (4.17)
	Acupuncture needle	45 (23.94)	45 (93.75)
Injured body site	Upper limb	4 (2.13)	0 (0.00)
	Lower limb	1 (0.53)	1 (2.08)
	Hand	139 (73.94)	41 (85.42)
	Eye	16 (8.51)	0 (0.00)
	Unknown	28 (14.89)	6 (12.50)
Wearing gloves	Wearing gloves	62 (32.98)	3 (6.25)
	Not wearing gloves	126 (67.02)	45 (93.75)
Source of exposure	Patient not tested/non-compliant	118 (62.77)	30 (62.50)
	Negative source	28 (14.89)	11 (22.92)
	HCV-related	2 (1.06)	0 (0.00)
	HBV-related	17 (9.04)	2 (4.17)
	Syphilis-related	18 (9.57)	5 (10.42)
	HIV-related	3 (1.60)	0 (0.00)
	Other	2 (1.06)	0 (0.00)
Reported within 24 h	No	1 (0.53)	0 (0.00)
	Yes	187 (99.47)	48 (100.00)
Medication use	No	150 (79.79)	42 (87.50)
	No (immune due to antibodies)	12 (6.38)	2 (4.17)
	Yes	26 (13.83)	4 (8.33)
Completion of 6-month follow-up	No	133 (70.74)	36 (75.00)
	Yes	55 (29.26)	12 (25.00)
Completion of testing procedure	No	133 (70.74)	36 (75.00)
	Yes	55 (29.26)	12 (25.00)
Occurrence of blood-borne infection	Uncertain	133 (70.74)	36 (75.00)
	No	55 (29.26)	12 (25.00)

### 3.2 Characteristics of TCM-related occupational exposures

A total of 48 TCM-related occupational exposure events were identified, accounting for 25.53% of all occupational exposures. The top three departments were the Department of Preventive Treatment of Disease (*n* = 15, 31.25%), Rheumatology (*n* = 11, 22.92%), and Rehabilitation Medicine (*n* = 4, 8.33%). The mean age of exposed individuals was 26.58 ± 6.88 years, and the majority had less than 5 years of work experience (83.33%). Females accounted for 66.67% of cases. Regarding occupational roles, interns represented the largest proportion (50.00%).

Most exposures occurred in inpatient wards (58.33%). The main exposure-related procedures were needle removal (52.08%) and acupuncture/electroacupuncture procedures (25.00%). All events were classified as sharps injuries, with acupuncture needles accounting for 93.75% of cases. The hands were the most frequently affected body site (85.42%). In terms of protective measures, 93.75% of exposures occurred without glove use. All cases received immediate wound irrigation and disinfection, and 100% were reported within 24 h. Regarding exposure sources, 62.50% were attributed to patients with unknown or uncooperative infection status. Only 25.00% of cases completed the 6-month follow-up and testing protocol, while the remaining 75.00% had indeterminate infection outcomes due to incomplete follow-up.

### 3.3 Comparison of TCM-related and non-TCM occupational exposures

Occupational exposure characteristics were compared between TCM-related and non-TCM-related events (Table 2). No statistically significant differences were observed in year distribution, sex, age, years of service, source of exposure, medication use, completion of the 6-month follow-up, completion of testing procedures, or blood-borne infection outcomes (all *p* > 0.05).

**Table 2:** Univariate comparison of occupational exposure characteristics between TCM-related and non-TCM-related groups.

Variables	Items	Whether TCM-related occupational exposure, *n* (%)		Total	Test statistic	*p*
		No (*n* = 140)	Yes (*n* = 48)			
Year	2022	27 (19.29)	6 (12.50)	33 (17.55)	1.316	0.725
	2023	39 (27.86)	16 (33.33)	55 (29.26)		
	2024	40 (28.57)	14 (29.17)	54 (28.72)		
	2025	34 (24.29)	12 (25.00)	46 (24.47)		
Sex	Female	108 (77.14)	32 (66.67)	140 (74.47)	2.063	0.151
	Male	32 (22.86)	16 (33.33)	48 (25.53)		
Age (years)		28.43 ± 7.21	26.58 ± 6.88		1.584	0.117
Years of service	<5	103 (73.57)	40 (83.33)	143 (76.06)	1.871	0.171
	≥5	37 (26.43)	8 (16.67)	45 (23.94)		
Occupational role	Physicians	60 (42.86)	39 (81.25)	99 (52.66)	21.233	<0.001
	Nurse	67 (47.86)	7 (14.58)	74 (39.36)		
	Other	13 (9.29)	2 (4.17)	15 (7.98)		
Location of occurrence	Other	16 (11.43)	0 (0.00)	16 (8.51)	-	0.001
	Treatment room	25 (17.86)	8 (16.67)	33 (17.55)		
	Operating room	15 (10.71)	0 (0.00)	15 (7.98)		
	Therapy room	5 (3.57)	7 (14.58)	12 (6.38)		
	Ward	63 (45.00)	28 (58.33)	91 (48.40)		
	Outpatient clinic	16 (11.43)	5 (10.42)	21 (11.17)		
Exposure stage	Needle removal/withdrawal	24 (17.14)	25 (52.08)	49 (26.06)	-	<0.001
	Instrument cleaning and handling	20 (14.29)	0 (0.00)	20 (10.64)		
	Surgical/suturing procedure	14 (10.00)	0 (0.00)	14 (7.45)		
	Injection/puncture procedure	49 (35.00)	2 (4.17)	51 (27.13)		
	Special exposure events	1 (0.71)	0 (0.00)	1 (0.53)		
	Environmental/residual sharp exposure	0 (0.00)	1 (2.08)	1 (0.53)		
	Electroacupuncture/acupuncture procedure	0 (0.00)	12 (25.00)	12 (6.38)		
	Blood/body fluid exposure	3 (2.14)	0 (0.00)	3 (1.60)		
	Sharp disposal/sharps handling	29 (20.71)	8 (16.67)	37 (19.68)		
Type of exposure	Sharps injury	117 (83.57)	48 (100.00)	165 (87.77)	-	0.001
	Non-sharps exposure	23 (16.43)	0 (0.00)	23 (12.23)		
Type of sharp instrument (*n* = 165)	Medical device	13 (11.11)	1 (2.08)	14 (8.48)	-	<0.001
	Surgical needle	15 (12.82)	0 (0.00)	15 (9.09)		
	Injection/blood collection needle	89 (76.07)	2 (4.17)	91 (55.15)		
	Acupuncture needle	0 (0.00)	45 (93.75)	45 (27.27)		
Injured body site (*n* = 160)	Hand	98 (83.05)	41 (97.62)	139 (86.88)	-	0.016
	Non-hand site	20 (16.95)	1 (2.38)	21 (13.13)		
Wearing gloves	Wearing gloves	59 (42.14)	3 (6.25)	62 (32.98)	20.834	<0.001
	Not wearing gloves	81 (57.86)	45 (93.75)	126 (67.02)		
Source of exposure	Unknown	88 (62.86)	30 (62.50)	118 (62.77)	4.523	0.104
	Positive	35 (25.00)	7 (14.58)	42 (22.34)		
	Negative	17 (12.14)	11 (22.92)	28 (14.89)		
Medication use	No	118 (84.29)	44 (91.67)	162 (86.17)	1.634	0.201
	Yes	22 (15.71)	4 (8.33)	26 (13.83)		
Completion of 6-month follow-up	No	97 (69.29)	36 (75.00)	133 (70.74)	0.564	0.453
	Yes	43 (30.71)	12 (25.00)	55 (29.26)		
Completion of testing procedure	No	97 (69.29)	36 (75.00)	133 (70.74)	0.564	0.453
	Yes	43 (30.71)	12 (25.00)	55 (29.26)		
Occurrence of blood-borne infection	Uncertain	97 (69.29)	36 (75.00)	133 (70.74)	0.564	0.453
	No	43 (30.71)	12 (25.00)	55 (29.26)		

In contrast, significant differences were identified in occupational role, location of occurrence, exposure stage, type of exposure, type of sharp instrument, injured body site, and glove use (all *p* < 0.05). TCM-related occupational exposures occurred predominantly among physicians (81.25%), whereas non-TCM-related exposures were more frequently reported among nurses (47.86%) and physicians (42.86%) (*p* < 0.001). TCM-related exposures also occurred more frequently in inpatient wards (58.33%) and therapy rooms (14.58%), whereas non-TCM-related exposures were more widely distributed across wards (45.00%), treatment rooms (17.86%), and operating rooms (10.71%) (*p* = 0.001).

Significant differences were also observed in exposure stage (*p* < 0.001). TCM-related occupational exposures mainly occurred during needle removal or withdrawal (52.08%) and electroacupuncture/acupuncture procedures (25.00%), whereas non-TCM-related exposures most commonly occurred during injection or puncture procedures (35.00%) and sharp disposal or sharps handling (20.71%). All TCM-related occupational exposures were classified as sharps injuries, compared with 83.57% in the non-TCM-related group (*p* = 0.001).

Among sharps injury events (*n* = 165), acupuncture needles accounted for the vast majority of devices involved in the TCM-related group (93.75%), whereas injection or blood collection needles were the most common devices in the non-TCM-related group (76.07%) (*p* < 0.001). Hand injuries were more common in the TCM-related group than in the non-TCM-related group (97.62% vs. 83.05%, *p* = 0.016). In addition, glove use differed significantly between groups, with non-use of gloves being substantially more frequent among TCM-related occupational exposures than among non-TCM-related occupational exposures (93.75% vs. 57.86%, *p* < 0.001).

### 3.4 Post-exposure management compliance and outcomes

Immediate wound irrigation and local disinfection were performed in all reported occupational exposure events, and nearly all events were reported within 24 h, indicating high compliance with immediate post-exposure management. In contrast, completion of the recommended 6-month follow-up and testing protocol was relatively low, with fewer than one-third of healthcare workers completing the full protocol. Consequently, infection outcomes remained indeterminate for most reported events because of incomplete follow-up. Detailed post-exposure management and outcome data are presented in Table 1.

## 4 Discussion

### 4.1 Epidemiological characteristics of reported occupational exposure events

This study analyzed 188 reported occupational exposure events from 2022 to 2025 in a tertiary teaching hospital. The results showed that needlestick and sharp injuries remained the predominant type of reported occupational exposure events. Female healthcare workers and those with less than 5 years of clinical experience accounted for a substantial proportion of reported exposure events, indicating that younger and less experienced healthcare workers represented an important group among reported occupational exposure cases. Previous studies have suggested that healthcare workers with limited clinical experience may account for a substantial proportion of occupational exposure events, possibly due to differences in procedural familiarity and adherence to standard precautions (11, 16). However, these factors were not directly evaluated in the present study, and the observed distribution of reported exposure events should be interpreted as characteristics of the reported cases rather than evidence that occupational exposure occurred more frequently in specific groups.

In addition, most reported occupational exposure events in this study occurred in inpatient wards and frontline clinical settings. Similar distributions have been reported in previous studies. Wong et al. (17), in a ten-year large-scale retrospective study, reported that approximately 59% of needlestick injuries occurred in wards, with junior staff contributing a substantial proportion of reported exposure cases. Furthermore, previous studies have suggested that insufficient occupational safety training and poor adherence to standard precautions may contribute to the occurrence of needlestick injuries (18). However, these factors were not directly evaluated in the present study. Therefore, prevention strategies should consider the characteristics of reported occupational exposure events among junior healthcare workers and frontline clinical settings, while further studies incorporating behavioral and organizational factors are needed.

### 4.2 Characteristics of TCM-related reported occupational exposure events

In this study, TCM-related occupational exposure events accounted for 25.53% of all reported exposure events. These events mainly occurred in departments where acupuncture-related practices were performed, including the Department of Preventive Treatment of Disease, Department of Rheumatology, and Department of Rehabilitation. Compared with non-TCM-related reported events, TCM-related events showed a more concentrated procedural pattern, with acupuncture needles accounting for the majority of exposure devices and all events being classified as sharp injuries.

Previous studies have also reported that acupuncture-related occupational exposures are closely associated with specific procedural steps, particularly needle handling and removal processes. Jiang et al. (15) found that acupuncture practitioners frequently experienced needlestick injuries, with many events occurring during needle removal procedures. Yu et al. (19) further reported that acupuncture needle injuries were concentrated during specific stages of clinical operation. These findings are generally consistent with the procedural characteristics observed in the present study.

The predominance of acupuncture needle-related injuries among TCM-related reported exposure events suggests that prevention strategies should consider the specific characteristics of acupuncture procedures. Potential approaches may include optimization of needle handling processes, reinforcement of standardized disposal procedures, and targeted occupational safety training. However, further studies incorporating procedure volume and exposure rates are needed to determine whether these procedural characteristics correspond to differences in occupational exposure risk.

### 4.3 Procedural characteristics and potential preventive implications

The present study identified differences in procedural characteristics between TCM-related and non-TCM-related reported occupational exposure events. TCM-related events mainly occurred during acupuncture-related procedures, particularly needle removal, whereas non-TCM-related events were more frequently associated with injection, puncture, and sharp device handling procedures. These findings indicate that different clinical practices may have different procedural contexts associated with reported exposure events.

In acupuncture practice, needle removal was identified as a high-risk procedure. This may be attributed to several factors, including decreased attention at the end of the procedure, continued exposure of needles on the patient’s body surface, and increased procedural complexity associated with the management of multiple needles. Jiang et al. (15) also confirmed that needle removal represents a high-risk stage for acupuncture-related needlestick injuries, with a considerable proportion of exposures occurring without glove use, thereby further increasing occupational risk. In contrast, invasive procedures such as injection and blood collection represented the primary exposure points in non-TCM-related practices. Negash et al. (20) reported that fatigue and high workload were important contributing factors for needlestick injuries, whereas strict adherence to standard precautions could significantly reduce exposure risk.

Furthermore, the present study found that the proportion of healthcare workers without documented glove use was significantly higher in the TCM group than in the non-TCM group (93.75% vs. 57.86%). This difference indicates variation in protective practices between TCM-related and non-TCM-related reported exposure events; however, the underlying reasons and their relationship with occupational exposure occurrence could not be determined from the present dataset. Previous studies have suggested that perceptions regarding glove use, procedural requirements, and adherence to standard precautions may influence protective practices during clinical procedures. In acupuncture practice, concerns regarding tactile sensation and acupoint localization have been reported as potential considerations affecting glove use; however, these factors were not directly assessed in this study. Therefore, prevention strategies should consider the specific characteristics of different clinical procedures, including optimization of needle handling workflows, reinforcement of sharp-device disposal practices for TCM-related procedures, and promotion of engineering controls, safety devices, and non-contact techniques for non-TCM-related procedures (21).

### 4.4 Deficiencies in post-exposure management and limitations in infection outcome assessment

The present study showed that immediate wound cleansing and local disinfection were documented for all reported occupational exposure events, and 99.47% of cases were reported within 24 h. These findings indicate that timely reporting and initial management procedures were generally well documented in this hospital. However, only 29.26% of reported exposure events completed the recommended 6-month follow-up and testing procedures, indicating limited completion of long-term follow-up after the initial exposure management process. Similar challenges regarding continuity of occupational exposure follow-up have also been reported in previous studies, suggesting that maintaining complete follow-up after initial exposure management remains an important component of occupational health practice (22).

Several practical factors may influence completion of long-term follow-up after occupational exposure events. In this study, some exposed individuals were interns, visiting trainees, or rotating staff who may have left the hospital after the exposure event, which could make continued follow-up more challenging within the institutional management system. Other factors, such as staff mobility and awareness of occupational exposure management, have been suggested as potential influences on follow-up completion; however, these factors were not directly assessed in the present study.

Among the reported exposure events with complete follow-up, no HBV, HCV, or HIV infections were confirmed. However, because 70.74% of reported exposure events did not complete the recommended follow-up and testing procedures, the occurrence of undetected blood-borne infections cannot be excluded, and the infection outcomes of all reported exposure events cannot be fully assessed. Therefore, these findings should be interpreted as “no confirmed infection outcomes were observed among cases with complete follow-up” rather than evidence that no infections occurred. Previous studies have indicated that accurate assessment of blood-borne infection outcomes after occupational exposure relies on complete follow-up systems (8).

Collectively, occupational exposure management should not only emphasize timely response and reporting procedures but also strengthen long-term follow-up and outcome surveillance. Strengthening comprehensive follow-up systems may improve the completeness of occupational exposure management and facilitate more accurate evaluation of post-exposure outcomes.

## 5 Limitations

This study has several limitations. First, as a single-center retrospective study, the sample size and data source were relatively limited, which may have introduced selection bias and limited the generalizability of the findings. Second, the study was based on the hospital occupational exposure reporting system, which records only reported occupational exposure events and does not routinely capture denominator information (e.g., the total number of healthcare workers, clinical procedures, or TCM-related procedures). Consequently, the incidence and occupational exposure risk could not be estimated. In addition, underreporting of occupational exposure events cannot be completely excluded because not all events may have been reported through the hospital reporting system. Therefore, the findings should be interpreted as descriptive characteristics of reported occupational exposure events rather than estimates of the true incidence or overall burden of occupational exposure within the hospital. Third, missing or incompletely recorded information for some variables may have affected the comprehensiveness of the analyses. Moreover, the relatively low follow-up completion rate limited the accurate assessment of long-term blood-borne infection outcomes following occupational exposure. Finally, multiple univariate comparisons were conducted as exploratory analyses without adjustment for multiple testing. Although these analyses were intended to describe differences in event characteristics rather than to establish independent associations, the possibility of type I error cannot be excluded, and statistically significant findings should therefore be interpreted with appropriate caution. Future multicenter prospective studies incorporating comprehensive denominator data and standardized follow-up systems are warranted to improve data completeness and enhance the reliability and generalizability of the findings.

## 6 Conclusion

Based on occupational exposure records from a tertiary hospital between 2022 and 2025, this study characterized the distribution of reported occupational exposure events and specifically examined the characteristics of TCM-related reported exposures. Sharp injuries remained the predominant type of reported occupational exposure events. Female healthcare workers and those with less than 5 years of clinical experience accounted for a substantial proportion of reported cases, particularly among frontline clinical activities.

TCM-related reported occupational exposure events were primarily associated with acupuncture and other TCM-specific clinical procedures. These events were characterized by a predominance of acupuncture needle-related sharp injuries and frequent occurrence during needle removal procedures. Differences in glove use were also observed between TCM-related and non-TCM-related reported exposure events. These findings suggest that TCM-related reported occupational exposure events have distinct procedural characteristics, mainly involving acupuncture needles, which may provide important considerations for developing procedure-specific prevention strategies.

Although immediate reporting and initial post-exposure management were well documented, completion of long-term follow-up remained insufficient among reported exposure events, indicating a need to improve continuity of post-exposure management. Therefore, occupational exposure prevention strategies should consider the device characteristics and critical procedural steps involved in TCM practices, with greater emphasis on procedure-specific prevention approaches. Strengthening closed-loop post-exposure management systems may improve follow-up completion and enhance occupational safety management among healthcare workers.

## Data Availability

The original contributions presented in the study are included in the article/supplementary material, further inquiries can be directed to the corresponding author.
